# Avian Physiology: Are Birds Simply Feathered Mammals?

**DOI:** 10.3389/fphys.2020.542466

**Published:** 2020-11-09

**Authors:** Colin G. Scanes

**Affiliations:** Department of Poultry Science, University of Arkansas, Fayetteville, AR, United States

**Keywords:** avian, domestication, ovary, oviduct, bursa, spleen

## Abstract

There are marked differences between the physiology of birds and mammals. These reflect the evolutionary distance between the two classes with the last common ancestor estimated as existing 318 million years ago. There are analogous organ systems in birds and mammals. However, marked differences exist. For instance, in the avian gastro-intestinal tract, there is a crop at the lower end of the esophagus. This functions both to store feed and for microbial action. The avian immune system lacks lymph nodes and has a distinct organ producing B-lymphocytes, namely the bursa *Fabricius*. The important of spleen has been largely dismissed until recently. However, its importance in both innate and specific immunity is increasingly recognized. There is a major difference between birds and mammals is the female reproductive system as birds produce large yolk filled eggs. The precursors of the yolk are synthesized by the liver. Another difference is that there is a single ovary and oviduct in birds.

## Introduction

The physiology of birds has attracted significant attention. A caveat is that much of the research on the physiology birds has been with domesticated birds, particularly chickens and ducks. The present communication discusses examples from the following systems where birds differ from mammals: gastrointestinal tract and specifically the crop and ceca, immune system and specifically the bursa *Fabricius* and spleen and female reproduction. Moreover, a series of questions are asked. It is noted that the physiology of birds reflects impacts of their evolutionary history, effects of domestication and the *sequentia* of flight.

### Evolutionary Relationships

Birds and mammals have been long separated. The *Synapsida* (mammals and extinct ancestors) and *Reptilia* (encompassing turtles, lizards, crocodiles, dinosaurs, and birds) diverged 318 million years ago (MYA) (Benton et al., 2015). Common features of all birds, or at least their ancestors, are the following: the ability to fly, the presence of feathers and the production of large yolky eggs with thick shells. Birds are much closer to lizards, snakes (last common ancestor – 256 MYA) and particularly crocodiles (last common ancestor – 247 MYA) than they are to mammals (Benton et al., 2015). Birds evolved from bipedal dinosaurs with the first true bird thought to have existed at the end of the Jurassic period/beginning of the Cretaceous period (Brusatte et al., 2015). Based on genomics, the last common ancestors were the following:

• *Palaeognathae* (ostriches, emus, tinamous, etc.) and the *Neognathae* (all other birds) – 113 MYA during Cretaceous period.
• *Neoaves* (virtually all birds today) and *Galloanseres* (ducks, geese, chickens, pheasants and their kin) 88 MYA during Cretaceous period.
• The ancestors of the major groups of birds including land birds and water birds diverged at about the time of the Cretaceous–Paleogene (K-Pg) boundary (66 MYA) with some diverging before and some immediately after (Brusatte et al., 2015).

An identifiable fossil land bird has been described from ∼62.5 million-year-old rocks (Ksepka et al., 2017). An “explosive” radiation of groups of birds occurred shortly after the K-Pg boundary (Ksepka et al., 2017).

### Domestication and Selection by Humans

There is clear evidence that domestication and later selection has influenced the genetics and phenotype of poultry. Chickens were domesticated from members of the *Gallus* genus beginning at least 8000 years ago (West and Zhou, 1988) with multiple domestication events in South Asia, Southwest China and Southeast Asia (Miao et al., 2017). The genetics of today’s chickens reflect genetics coming from red jungle fowl (*Gallus gallus*) together with introgressions from the green jungle fowl (*G. varius*) (Sawai et al., 2010) and the gray jungle fowl (*G. sonneratii*) presumably after domestication. Prior to scientific selection, there were also shifts in the genetics and hence physiology of poultry. For instance, based on studies with native chickens from Africa and Europe, chickens from different regions are genetically equipped for different environmental temperatures (Fleming et al., 2017). Breeds of domesticated poultry were recognized considerably over 100 years ago; the development of these reflecting genetic drift and hardiness within specific locals together human intervention.

#### Caveats

White Leghorns are frequently used as a surrogate for all chickens or even all birds (e.g., Roth and Lind, 2013; Fallahsharoudi et al., 2015; Løtvedt et al., 2017) but the sources of White Leghorns vary as does their genetics. Another issue is that commercial breeding of broiler chickens, laying chickens, turkeys, and ducks is closely held within primary breeding companies with the genetics “protected.” The lines are subjected to intense selection focusing on commercially important parameters such as growth rate, egg production, and efficiency. An example of the changes in genetics is the over four-fold increase in growth rates in meat-type chickens (Table 1). Similarly, increases in growth rate have been reported by Havenstein et al. (1994, 2003) comparing, respectively, 1991 and 2001 meat-type chickens with random bred chickens. There have continued to be improvements in growth rate since 2005. The genetics of the birds differ even for lines having the same name due to selection and use of different grandparent lines. This is very different from situation with inbred rodent lines.

**TABLE 1:** Effect of genetic selection of growth in meat-type chickens (Zuidhof et al., 2014).

**Average daily gain g d^–1^**	**Chickens**	
**Between days**	**Control^#^**	**2005 commercial meat-type**
1–7	4.6 ± 0.15^*a*^	15.9 ± 0.15^*b*^
22–28	15.3 ± 0.65^*a*^	81.9 ± 0.65^*b*^
36–42	20.6 ± 1.04^*a*^	99.6 ± 1.04^*b*^
50–56	23.0 ± 1.65^*a*^	101.1 ± 1.65^*b*^

*^*a,b*^Different superscript letters indicate difference from control *p* < 0.05. ^#^Random bred – not subject to scientific selection.*

### Impact of Domestication and Selection by Humans

It is increasingly recognized that successful domestication was accompanied by shifts in genetics, and hence physiology. Domestication alone or with later selection was associated with shifts in the responses to stress including within the hypothalamic pituitary axis (Fallahsharoudi et al., 2015; Løtvedt et al., 2017). Differences in the stress physiology have been reported between chickens of a major egg laying breed (White Leghorns) and wild Red Jungle fowl (*Gallus gallus*) with depressed basal circulating concentrations of pregnenolone and dehydroepiandrosterone (DHEA) together with circulating concentrations of corticosterone following restraint in domesticated chickens (Fallahsharoudi et al., 2015). In addition, there is increased expression of the following stress related genes under both basal or stress conditions in the hypothalamus of domesticated chickens: *CRHR1*, *AVP*, and *GR* (Løtvedt et al., 2017). There have shifts in the eye after domestication and selection with, for example, red jungle fowl having greater optical sensitivities at low light intensities than White Leghorn chickens (Roth and Lind, 2013). Moreover, pea-comb mutation is related to SOX5 (Wright D. et al., 2009) leads to reduced comb and wattle size and, thereby, leading to reduction in susceptibility to lesions following freezing temperatures (reviewed: Wright D. et al., 2009).

Shifts in supposedly “domestication related” genes have been reported for yellow skin color (β-carotene dioxygenase 2) and thyroid-stimulating hormone receptor (*TSHR*) (a missense mutation from glycine to arginine) and wild-type allele (Rubin et al., 2010; Karlsson et al., 2015). However, these mutations appear to occurred within the past 500 years rather than at the time of domestication (Garland Flink et al., 2014).

### Differences in Body Weight Between Mammals and Birds

Mammals and birds have the same organ systems but there are differences (these being discussed below). Table 1 shows relative weights for critical organs in mammals and birds together with blood flow. The relative weights of heart, liver and kidneys differ between mammals and birds (Table 1) being increased for heart (increased by 3.54-fold), liver (decreased by 47.7%) and kidneys (increased by 56.1%) in birds. Spleen relative weights are markedly lower (73.9%) in birds than mammals (Table 2). Blood flow is similar between mammals and that in the, albeit low number of birds examined (Table 1). **Question 1: What accounts for the higher relative weights of the heart, kidneys and liver? Question 1: Are they related to lower efficiency of avian systems or to specific needs of, respectively, flight, uric acid excretion and egg production?**

**TABLE 2:** Comparison of the relative weights of and blood flow to major organs in mammals and birds.

**Organ**	**Relative organ weight%^*P*^**		**Blood flow ml min^–1^ g^–1^**	
	**Mammals**	**Birds**	**Mammals^*Q*^**	**Birds [duck^*R*^] (chicken^*S*^)**
Brain	0.999 ± (6) 0.321	1.469 ± (8) 0.426	0.88 ± (8) 0.11	0.84 [0.84]
Heart	0.706 ± (6) 0.052	2.497 ± (11) 0.299***	3.29 ± (7) 0.85	3.94 [2.69] (5.28)
Liver	2.831 ± (6) 0.378	1.481 ± (11) 0.212**	0.42 ± (6) 0.15	0.62 [0.58] (0.67)
Kidney	0.620 ± (6) 0.095	0.968 ± (11) 0.090*	3.83 ± (7) 0.36	4.43 [1.08] (7.78)
Spleen	0.336 ± (5) 0.038	0.0877 ± (4) 0.0115***	2.02 ± (7) 0.63	4.16 [5.56] (2.77)

*p < 0.05, **p < 0.01, ***p < 0.001 compared to mammals. ^*P*^Data is the mean of the means of mammalian or avian orders (*n* = the number of orders) ± SEM]. ^*Q*^Based on mean ± (*n* = number of species) SEM. ^‡^Data from dogs (Li et al., 1989), Mongolian gerbil (Matsumoto et al., 1982), mouse (Wang et al., 1993), rabbit (Neutze et al., 1968), pigs (neonatal Undar et al., 1999; Thein et al., 2003), rats (Alexander et al., 1972; Ishise et al., 1980; Sakanashi et al., 1987; Adán et al., 1994), rhesus monkey (infant: Behrman and Lees, 1971), and sheep (fetal: Tan et al., 1997) (neonatal: Alexander et al., 1972). ^*R*^Data from Jones et al. (1979) and Kaul et al. (1983). ^*S*^Sapirstein and Hartman (1959); Boelkins et al. (1973), and Merrill et al. (1981).

## Gastrointestinal Tract

The gastrointestinal tract of birds shows close similarities to that of mammals with, for instance, an esophagus for the passage of ingesta from the mouth to the equivalent of the stomach and a small intestine made up of duodenum, jejunum and ileum where much enzymatic digestion and virtually all of absorption occurs and a colon (large intestine). Moreover, the liver and pancreas play similar roles supplying, respectively, bile with bile salts and pancreatic juice with digestive enzymes. There are also differences including the following, using chickens and ducks as exemplars:

1. The absence of teeth and hence chewing.
2. The presence of the crop as an outgrowth of the lower esophagus.
3. The separation of the enzymatic and muscular aspects of the stomach into the proventriculus and gizzard.
4. The presence of two large ceca.
5. The small size of the colon.
6. The presence of a common exit for urine and feces.
7. The retrograde flow of ingesta with urine from the cloaca through the colon.

Two avian features will be considered in more detail, namely the crop and ceca.

### Crop

Based on studies in chickens and turkeys, the crop can act as a storage organ for feed. While there is little feed in the crop during the day, ingesta are present in the crop throughout the scotophase (e.g., Scanes et al., 1987; Buyse et al., 1993; Johannsen et al., 2005) when feeding is not occurring (references) (Table 3). The amount of feed in the crop progressively declines during the night (e.g., Scanes et al., 1987; Buyse et al., 1993; Johannsen et al., 2005) (Table 3). Thus, the situation appears to be that the chickens and turkeys gorge in the late afternoon (Scanes et al., 1987; Buyse et al., 1993) with the feed stored in the crop to be released during the nocturnal fast. Similarly, there is storage of feed in meal fed meat-type sexually immature female chickens (broiler breeder pullets) with approximately double stored when fed on alternate days (de Beer et al., 2008). A case can also be made for the proventriculus/gizzard being a site for feed storage (Table 3).

**TABLE 3:** Changes in the crop attributes during the scotophase.

	**Time relative to the beginning**		
	**of the scotophase (night/darkness)**		
	**0→+1 h**	**+5 h**	**+9→10 h**
**Laying hen**^*p*^			
Crop contents g	46.0 ± 5.4^*c*^	18.1 ± 4.1^*b*^	1.9 ± 1.0^*c*^
Soluble carbohydrate% of dry wt. of contents	17.1 ± 0.5^*a*^	18.3 ± 4.2^*a*^	8.5 ± 1.1^*b*^
Proventriculus/gizzard contents g	28.8 ± 2.6^*c*^	21.4 ± 1.3^*b*^	15.5 ± 1.5^*a*^
**Young meat-type chicken^*q*^**			
Crop contents g	28.4 ± 6.9^*c*^	11.6 ± 3.5^*b*^	0.2 ± 0.08^*c*^
Proventriculus/gizzard contents g	22.2 ± 3.0^*b*^	11.0 ± 1.9^*a*^	7.4 ± 1.5^*a*^
**Turkey poult**^*r*^			
Contents g	22.2 ± 2.4^*a*^	22.4 ± 3.9^*a*^	13.2 ± 2.3^*b*^
Moisture%	39.4 ± 2.3^*a*^	29.5 ± 1.4^*b*^	29.7 ± 2.1^*b*^
pH	5.9 ± 0.1^*a*^	5.0 ± 0.2^*b*^	5.0 ± 0.2^*b*^
Lactic acid μMoles g^–1^	13.4 ± 4.5^*a*^	93.3 ± 17.4^*b*^	98.4 ± 11.4^*b*^
Caproic acid (C6) μMoles g^–1^	0.17 ± 0.02^*a*^	0.87 ± 0.28^*b*^	1.33 ± 0.06^*b*^
Valeric acid (C5) μMoles g^–1^	0.13 ± 0.04^*a*^	0.11 ± 0.03^*a*^	0.83 ± 0.11^*b*^

*^*a,b,c*^Different superscript letters indicate difference *p* < 0.05. ^*p*^Scanes et al. (1987). ^*q*^Buyse et al. (1993). ^*r*^Johannsen et al. (2005).*

Both the crop developed and the stomach separated into two distinct anatomical features (the gizzard and proventriculus); occurring during avian evolution and with the arrangement has been retained in multiple taxonomic groups.** Question: What is the selective advantages if these?**

The ability of the hoatzin (*Opisthocomus hoazin*) to ferment plant materials is well established (Grajal et al., 1989) with the presence of rumen-like methanogens confirmed (Wright A.D. et al., 2009). Some consider that the hoatzin is the only avian fore-gut fermenter (e.g., Wright A.D. et al., 2009). However, there is evidence for crop fermentation with the extended nocturnal storage of ingesta (see Table 3). During the night when chickens and turkeys do not eat (reference), there is a gradual release of ingesta (see Table 3). There are also decreases in soluble carbohydrate (Table 3) (laying hen: Scanes et al., 1987) and increases in the concentrations of organic acids, predominantly lactic acid/lactate (Table 3) (turkey poult: Johannsen et al., 2005). There was not evidence for production of the major volatile fatty acids (VFAs): acetic acid/acetate, propionic acid/propionate or butyric acid/butyrate (turkey poult: Johannsen et al., 2005). What is not clear is whether and, if so, the rate to which, lactic acid/lactate and other fatty acids are absorbed from the crop? Interestingly, there is evidence that the crop plays a role in calcium absorption in laying hens with reduced egg production and serum calcium concentrations following cropectomy (Stonerock et al., 1975).

There are also marked increases in the lactic acid/lactate concentrations of the ingesta from the crop to proventriculus/gizzard and along the small intestine (domestic goose: Clemens et al., 1975). This may reflect microbial fermentation or anaerobic metabolism by gut tissues. Given that there is extended storage of feed in the proventriculus/gizzard during the night (Table 3), it is not clear whether there is also fermentation in the proventriculus/gizzard. **Question: How much lactic acid and other potential nutrients absorbed from the crop?**

### Ceca

The ceca are major sites of fermentation with production of VFAs with the concentration of VFAs of 107.3 ± (3) 4.1 μMoles g^–1^ in the cecal contents (laying hen: Annison et al., 1968). Table 4 summarizes the contribution of individual VFAs in the colon contents of chickens. Acetic acid/acetate is the dominant VFA. The VFA concentrations of cecal content concentrations increase following feeding rising from ∼20 μmoles ml^–1^ 2 h after feeding to ∼70 μmoles ml^–1^ 8 h after feeding (domestic goose: Clemens et al., 1975). The ceca are also the major site of methane production by, at least geese; with methane production being reduced by 91% in cecectomized geese (Chen et al., 2003) (Table 5). In contrast, nitrous oxide emission from geese is not influenced by cecectomy (Chen et al., 2003) (Table 5). Addition of alfalfa can reduce methane production by over 70% in Muscovy ducks, mule ducks and white Roman geese (Chen et al., 2014). Interestingly, there was no overall effect of addition of antibiotics to feed on VFA production by chickens (Kumar et al., 2018).

**TABLE 4:** Proportion of individual VFAs generated in the ceca of chickens.

	**Young meat-type chickens^*X*^**	**Adult female chickens^*Y*^**
Acetic acid/acetate (C2)	76.9	51.6 ± (10) 2.8
Propionic acid/propionate (C3)	5.9	26.7 ± (10) 1.9
Butyric acid/butyrate (C4)	17.5	9.2 ± (10) 0.8
Isobutyric acid/isobutyrate (C4)	ND	1.3 ± (10) 0.4
Isovaleric acid (C5)/2 methyl butyric acid	ND	1.8 ± (10) 0.5
Valeric acid/valerate (C5)	ND	2.0 ± (10) 0.4

*^*X*^Kumar et al. (2018). ^*Y*^Calculated from data in Annison et al. (1968) for laying hens. ND, not detected.*

**TABLE 5:** Generation by methane and nitrous oxide by poultry.

**Species**	**Methane generated**	**Nitrous oxide generated**
	**mg bird^–1^ day^–1*A*^**	**mg bird^–1^ day^–1*A*^**
Muscovy duck	26.4	
Mule	17.4	
Domesticated goose	11.4	0.45^*B*^
	kg head^–1^ life cycle^–1*BC*^	kg head^–1^ life cycle^–1*BC*^
Broiler chickens	1.59 × 10^–5^	3 × 10^–8^
Village chickens	8.48 × 10^–5^	1.635 × 10^–5^
Geese	1.5 × 10^–3^	4.90 × 10^–5^

*^*A*^Chen et al. (2014). ^*B*^Chen et al. (2003). ^*C*^Wang and Huang (2005).*

What is not clear is whether and, if so, the rate to which, VFAs are absorbed from the ceca? The concentrations of VFA in the ingesta have been reported to decrease in the colon and cloaca (Clemens et al., 1975). This arguably indicates absorption. The overall importance of the ceca is not supported by the lack of effect of cecectomy on growth rates in geese (Chen et al., 2003).

**Question 1: What are the rates of absorption of VFAs and other nutrients from the ceca? Question 2: Is this physiologically relevant and, if not, why are the ceca so large?**

## Immune System

There are similarities between the avian and mammalian immune system with the presence of both innate and both T and B cell mediated specific immune responses and the presence of the thymus. However, there are marked differences between the organization of the immune system in the two classes including the following in birds:

1. The presence of the bursa Fabricius – the primary immune organ producing B cells in birds.
2. The lack of lymph nodes in birds.

**Question 1: Are the structures that have yet to be described that are essentially “lymph nodes”? Question 2: What have birds to replace the functions of the lymph nodes?**

Until recently, the avian spleen was largely ignored. Indeed, John (1994) concluded that the avian spleen was “little-studied” by avian physiologists. This is no longer the case with substantial interest by immunologists. Examples of advances in our understanding of the spleen are discussed below.

### Bursa Fabricius

The avian bursa Fabricius played an important role in the development of understanding of immune functioning. Antibody formation is greatly reduced in chickens bursectomized at 2 weeks old (Glick et al., 1956; Ewert and Eidson, 1977). Birds that were bursectomized at 60 h of embryonic development have immunoglobulin Ig producing B cells but do not exhibit an ability to generate a specific antibody response (Mansikka et al., 1990).

### Spleen

#### Anatomy

The avian spleen is smaller than the mammalian organ (see Table 1) (John, 1994). In birds, the spleen is surrounded by a connective tissue capsule (Kannan et al., 2015). The avian spleen can be considered composed of two tissues: red pulp (with blood containing sinusoids with cords of lymphocytes, macrophages, granulocytes, plasma cells, and mast cells) and white pulp (lymphoid tissue composed of young lymphoblasts, lymphocytes, follicular dendritic cells, and reticular cells) (chicken: Oláh and Glick, 1982; Kannan et al., 2015; reviewed: Powers, 2000). There are unmyelinated nerve fibers present in the ellipsoids (Oláh and Glick, 1982). Central artery is surrounded by ellipsoids (Oláh and Glick, 1982). The venous drainage from the spleen passes to the hepatic portal vein (Powers, 2000). There is evidence for a blood-spleen barrier in birds (domestic duck: Sun et al., 2019).

#### Roles of the Avian Spleen

The roles of the avian spleen are considered as the following:

1. As a lymphoid organ
2. Phagocytosis of senescent or damaged erythrocytes (Powers, 2000).

However, the avian spleen does not appear to be a temporary store of erythrocytes (Powers, 2000). **Question 1: If the spleen does not act as a temporary storage site for erythrocytes, is there an alternate system?**

#### Stressors and the Spleen

Spleen weights in birds are depressed by stress (see Table 6). This is irrespective of whether the stress is mimicked by the administration of corticosterone (Mehaisen et al., 2017) or represents transportation stress (Zhang et al., 2019) or protein deficiency (Adrizal et al., 2019). It is not surprising given the ability of corticosterone to depress the weight of the spleen (Mehaisen et al., 2017; also see Table 4) that the *MC2-R gene* is expressed in the chicken spleen (Takeuchi and Takahashi, 1998). Moreover, heat stress markedly increased the incidence of lesions in the spleen of broiler chickens (Aguanta et al., 2018). Similarly, corticosterone decreases the weights of the primary immune organs, thymus and bursa Fabricius, in birds (e.g., young chickens: Mehaisen et al., 2017). Spleen weights are also decreased following exposure to toxicants (Aflatoxin B_1_: Grozeva et al., 2017; Ochratoxin A: Khan et al., 2019; T-2 toxin: Hayes and Wobeser, 1983) (see Table 6). In contrast, spleen weights were increased after viral challenges or administration of *E. coli* lipopolysaccharide (see Table 6) (Boa-Amponsem et al., 1999; Zhang et al., 2017; Bai et al., 2019; Yang et al., 2019).

**TABLE 6:** Effect of challenges on spleen weights or relative spleen weights (data is expressed as the percentage of the control ± SEM).

**Treatment**	**Species**	**Control**	**Treated**	**Calculated from reference**
Corticosterone^*n*^	Young chickens	100 ± 5^*b*^	68 ± 12^*a*^	Mehaisen et al. (2017)
Transportation stress^#^	Young chickens	100 ± 3^*b*^	88 ± 3^*a*^	Zhang et al. (2019)
Low protein feed	Young chickens	100 ± 6^*b*^	76 ± 6^*a*^	Adrizal et al. (2019)
Aflatoxin B_1_	Turkey poults	100 ± 15^*b*^	71 ± 7^*a*^	Grozeva et al. (2017)
Ochratoxin A^#*p*^	Young chickens	100 ± 9^*b*^	52 ± 4^*a*^	Khan et al. (2019)
T-2 toxin^#*q*^	Mallard ducks	100 ± 2	83 ± 3	Hayes and Wobeser (1983)
LPS^*r*^	Young chickens	100 ± 2^*a*^	119 ± 2^*b*^	Yang et al. (2019)
Low energy feed and LPS	Yong ducks	100 ± 6^*a*^	112 ± 6^*b*^	Bai et al. (2019)
Marble spleen disease virus^#^	Young chickens	100 ± 5^*a*^	136 ± 6^*b*^	Boa-Amponsem et al. (1999)
Chicken infectious anemia virus^*s*^	Young (SPF) chickens	100 ± 13^*a*^	161 ± 31^*b*^	Zhang et al. (2017)
Marek’s disease virus^*s*^		100 ± 13^*a*^	132 ± 14^*b*^	Zhang et al. (2017)

*^*a,b*^Different superscript letters indicate difference from control *p* < 0.05. ^#^Relative spleen weights. ^*n*^Daily i.m. injections of 0.5 mg kg^–1^ for 7 days. ^*p*^Subcutaneous administration. ^*q*^Treatment for 2 weeks. ^*r*^*E. coli* lipopolysaccharides injected i.p. on alternate days × 4. ^*s*^After 9 days.*

#### Effects of Splenectomy

There is evidence that the avian spleen has both positive and negative effects on immunity. Splenectomy decreased primary immune response (the titer of antisera) after intravenous challenge with sheep red blood cells (Hippeläinen and Naukkarinen, 1990). Paradoxically, splenectomy improved the response of turkey poults challenged with hemorrhagic enteritis virus (HEV); decreasing the incidence of hemorrhagic diarrhea and mortalities (Ossa et al., 1983).

#### The Avian Spleen and Immune Functioning

The avian spleen plays important roles in both innate and specific immune responses. *STING* (stimulator of interferon genes) is expressed in the spleen (chicken: Ran et al., 2018). Infection of specific pathogen-free chickens with Newcastle Disease Virus was followed by increased expression of STING together with that of interferon (INF)-α, INF-β, and Interferon Regulatory Factor 7 (IRF-7) in the spleen (Ran et al., 2018). Rous sarcoma virus (RSV) infection of susceptible chickens is followed by increased expression of pro-inflammatory cytokines such as in spleen including interleukin (IL) 8 and IL 10. Moreover, there is marked expression of the Th1 cytokines IFN-γ and TNF-α in the spleen (Khare et al., 2019). In pigeons, infection with Newcastle disease viruses increases expression of *RIG-I*, *IL-6, IL-1*β, *CCL5*, and *IL-8* genes in the spleen (Xiang et al., 2019). LPS challenge increased antioxidant capacity with elevated malondialdehyde (MDA) concentrations in chicken spleen (Yang et al., 2019). There is high expression of toll-like receptor (TLR) 5 in the spleen and peripheral blood mononuclear cells (pigeon: Xiong et al., 2018). In the presence of flagellin (from Gram-negative bacteria), there is increased expression of TLR5, interleukin (IL)-6, IL-8, CCL5, IFN-γ and NF-κB by pigeon splenocytes (pigeon: Xiong et al., 2018). Administration of a synthetic ligand for TLR21 increased expression of IFNα but decreased that of IL-6 in the chicken spleen (Sajewicz-Krukowska et al., 2017). Chickens infected with Marek’s disease virus (Gallid herpesvirus 2) have more γδ T cells in their spleens (Laursen et al., 2018). Spread of Marek’s disease virus (MDV) to the spleen and thymus is delayed in Ig heavy chain J gene segment knockout (JH-KO) chickens lacking mature and peripheral B cells (Bertzbach et al., 2018). *Escherichia coli* infection is accompanied by decreased splenic expression of antibacterial nucleotide-binding oligomerization domain-like receptor (NLR) pyrin domain containing 3 (NLRP3) (Li et al., 2018). **Question 1: Is there redundancy such that the spleen is notnecessary in birds?**

## Female Reproduction

The avian egg is large with a yolk filled ovum, surrounded by egg white, membranes and a shell composed of calcium carbonate. Yolk is composed of the following:

• Water – ∼50%
• Solids – ∼50% composed of the following:

○ Low-density fraction (∼65% of yolk solids)
○ Granules (∼25% of yolk solids) composed of the following:

∎ Lipovitellin (a lipoprotein formed by the cleavage of vitellogenin in the oocyte)
∎ Phosvitin (a phosphoprotein formed by the cleavage of vitellogenin in the oocyte)

○ Water soluble or proteins in the aqueous fraction – the livetins (∼10% of yolk solids) composed of the following:

∎ α Livetins – Synonymous with blood serum albumen
∎ β Livetins – Synonymous with blood serum α_2_-globulin presumably containing transport proteins
∎ γ Livetins – Synonymous with blood serum γ -globulin specifically immunoglobulin Y (IgY) (equivalent to IgG in mammals).

The yolk precursors, vitellogenin (VTG) and very low density lipoproteins (VLDL), are synthesized in the liver under the estrogen stimulus (Deeley et al., 1975; reviewed Loh et al., 2011). They pass to the ovary via the circulatory system and their transport into the oocyte mediated by the receptor VLDL/VTG receptor (VLDL/VTGR) (chickens: Steyrer et al., 1990; Stifani et al., 1990; Barber et al., 1991; Bujo et al., 1994). Restricted ovulator chickens have a mutation in the VLDLR//VTGR and exhibit markedly reduced follicular development with elevated circulating concentrations of phospholipids, triglyceride and cholesterol (Elkin et al., 2003, 2012). However, there was still yolk deposition in the presence of the mutant VLDLR (Elkin et al., 2003, 2012). This suggests the existence of an alternate mechanism for deposition of the yolk precursors. **Question 1: Are there alternate mechanisms for transport of yolk precursors into the oocyte?**

There is little information on the transport of livetins into the developing oocyte. The γ livetins are almost exclusively (97.7%) IgY (Hamal et al., 2006; Agrawal et al., 2016). **Question 2: What is the mechanism for the transport of IgY into the oocyte? Question 3: What is the mechanism for selection of IgY versus IgM and IgA? Question 4: To what extent does this reflect limits on molecular size for proteins passing into the interstitial space?** The concentrations of albumen and γ-globulin in the interstitial space of chickens were greater than those of α-globulin and β-globulin when expressed as a percentage of vascular concentrations (calculated from data in Peltonen and Sankari, 2011). The granulosa cell layer and the tight junctions between cells may act in an analogous manner differentially permitting some, but not other, proteins to transit. There is much higher expression of the tight junction protein, occludin, by granulosa cells from smaller white follicles than large yellow follicles and being absent in preovulatory follicles (Schuster et al., 2004). The transport of cations into the yolk has received little attention. It is reasonable to assume that transport of calcium into the yolk occurs along with vitellogenin with calcium bound to the phosphate moieties. Sodium concentrations in the aqueous fraction are 44.6 mEquiv. L^–1^ (Gilbert, 1971; Grau et al., 1979). This is markedly higher than reported intracellular concentrations of sodium (erythrocyte: 13.6 mEquiv. L^–1^) and lower than the plasma concentration (Miseta et al., 1993; reviewed: Scanes, 2015). **Question 5: What are the mechanisms for sodium and other cations transport across the oocyte membrane?** At least some of the sodium is likely to enter along with VTG during endocytosis but then why isn’t sodium pump out?

In most birds, there is a single ovary (the left) and the left Müllerian duct develops into an organ called the oviduct. However, in a few species such as kiwis, there are paired ovaries and oviducts (Kinsky, 1971). The oviduct is made up of the following: infundibulum, magnum, isthmus, shell gland or uterus and vagina. Neither the oviduct nor uterus are equivalent to their name-sakes in mammals although both are derived from the Müllerian duct. The ovary is larger than that of mammalian ovaries with the ovary having a relative weight in sexually mature chickens of 2.42 ± (number of studies = 13) SEM 0.22% of body weight and sexually mature ducks 2.45% of body weight (see Table 7). This is due to the yolk accumulating in the oocyte within the follicle with the yolk precursors synthesized in the liver (see above).

**TABLE 7:** Relative weights of the ovary and oviduct in ducks and chickens together with blood flow [data is shown as mean ± (number of studies) SEM].

**Tissue**	**Ovary**	**Oviduct**	**References**
**Relative weight%**			
Sexually immature chickens	0.046 ± (4) 0.15^*a*^	0.046 ± (2) 0.24^*a*^	Maurice et al. (1982); Sun et al. (2006); Martínez et al. (2015); Dunn et al. (2017)
Sexually mature ducks	2.45	2.47	White et al. (1978)
Sexually mature chickens	2.42 ± (13) 0.22^*b*^	4.165 ± (7) 0.95^*b*^	Brody et al. (1984); Kwakkel et al. (1995); Joseph et al. (2000); Sun et al. (2006); Chen et al. (2007); Emiola et al. (2011); Pishnamazi et al. (2014); Saki et al. (2014); Sun et al. (2015); Hassan et al. (2016); Youssef et al. (2016); Nassar et al. (2017)
**Blood flow ml min**^–1^			
Sexually mature chickens	5.05 ± (3) 1.12	24.0 ± (6) 4.37	Boelkins et al. (1973); Moynihan and Edwards (1975); Niezgoda et al. (1979); Scanes et al. (1982); Hrabia et al. (2005); Rząsa et al. (2008)

*^*a,b*^Different superscript letters indicates difference *p* < 0.05.*

The relative weights of avian ovaries and oviducts are shown in Table 7. There are marked increases in both between sexually immature and mature female chickens with increases of 52-fold for the ovary and 91-fold for the oviduct (Table 7). There is high blood flow to both the ovary and oviduct (Table 7). There are also differences in blood flow to regions of the oviduct with higher blood flow to the magnum 9.56 ± (6) 2.49 ml min^–1^ and shell gland 10.1 ± (6) 2.42 ml min^–1^ than the infundibulum 1.01 ± (5) 0.10 ml min^–1^, isthmus 2.47 ± (6) 0.40 ml min^–1^ and vagina 0.96 ± (2) 0.20 ml min^–1^ (Boelkins et al., 1973; Moynihan and Edwards, 1975; Niezgoda et al., 1979; Scanes et al., 1982; Hrabia et al., 2005; Rząsa et al., 2008).

There are several studies on the effects of neurotransmitters on blood flow to the ovary and oviduct in birds. Histamine increased blood flow to stroma, small white follicles, large yellow follicles and post-ovulatory follicles together with the infundibulum and shell gland. In addition, histamine increased cardiac output (Hrabia et al., 2005). Serotonin induces a transient decrease (after 1 min) in blood flow to small white follicles and F4 and F5 large yellow follicles and to the shell gland with blood flow restored to at least pretreatment after 5 min (Rząsa et al., 2008). Similarly, prostaglandin F_2__α_ decreases blood flow to the large yellow follicles (Scanes et al., 1982). What is missing, are studies of the role of the nervous systems. **Question 1: What are the roles of the nervous system in the control of ovarian and oviductal functioning? Question 2: The control of blood flow to ovary and oviduct warrants further attention. Question 4: What is not clear is the extent blood flow reflect the metabolic requirements of a tissue or is blood flow a driving force dictating or restricting the metabolism of tissues?**

There are substantial loads placed on the female bird in synthesizing the proteins of egg white proteins, the membranes and the shell. In female birds, calcium is stored in a short-term basis in medullary bone in long bones. Calcium is mobilized from this storage in an attempt to balance the outflow of calcium in the uterus (shell gland) forming the egg. **Question: How is calcium mobilized from medullary bone?** In addition to parathyroid hormone, and 1,25 dihydroxy vitamin D_3_ (Castillo et al., 1979), there appears to be an involvement of other mechanisms. There is increased expression of receptor activator of nuclear factor-kB (RANK) and fibroblast growth factor (FGF23) in medullary bone of hens peaking at the end of egg calcification and the end of calcium mobilization from the medullary bone (Gloux et al., 2020). There was also increased expression of solute carrier family 20 member 1 (SLC20A1) and member 2 (SLC20A2) (Gloux et al., 2020). Moreover, circulating concentrations of phosphate are elevated in laying hens passively immunized against FGF23 (Ren et al., 2019).

The mechanism for calcium transfer to the shell has received considerable attention. It is mediated at least in part by calbindin D 28K (formerly known as vitamin D-dependent calcium-binding protein) (Bar et al., 1996). Uterine expression of calbindin D 28K is increased by estrogens if 1,25 dihydroxy vitamin D3 is present (Nys et al., 1992). Androgens potentiate the ability of estrogens to increase expression of calbindin D 28K in the uterus during sexual maturation (Nys et al., 1989). Moreover, there are shifts in uterine gene expression of as the ovum passed down the oviduct with, as might be expected, increased expression of calbindin D 28K and transient receptor potential vanilloid channel type 6 (TRPV6) when the egg is being calcified (Nys et al., 1989; Yang et al., 2013). Concentrations of CaBP-D28k protein in the uterine mucosa are depressed in the presence of either interleukin-1β and interleukin-6 *in vitro* (Nii et al., 2018). **Question 1: What are other mechanisms, if any, for calcium transport?**

## Conclusion

In avian physiology, there are a series of assumptions employed that may or may not be valid. Examples of these assumptions include the following:

• Domesticated birds provide little or no information about wild birds as the former have been subjected to intensive anthropomorphic selection.
• The contrary view is that the physiology of one species of bird, domesticated or wild, are readily transferable to another.

The debate between these views is accentuated by some rigidity of those with either an ornithological or poultry orientation. This situation is confounded by the lack of common meetings, departments and education. The differences in education include the following:

• The lack of courses (particularly at the graduate level) experienced by poultry physiologists on wild birds and on the ungirding principle of biology, namely evolution.
• There is a corollary with appreciation for poultry lacking in those studying wild birds.

Another erroneous assumption is that birds are merely “feathered” mammals with a few specific differences related, for instance, to flight and production of large yolky eggs. Instead, the physiology of birds reflects their long evolutionary history.

Finally, to adapt the quotation from both George Santayana and Winston Churchill, “*Those who fail to learn from or even read the literature including the older literature are doomed to repeat the studies and not advance science.*”

## Author Contributions

This is solely the work of CS.

## Conflict of Interest

The author declares that the research was conducted in the absence of any commercial or financial relationships that could be construed as a potential conflict of interest.

## References

[B1] Adán C. Ardévol A. Remesar X. Alemany M. (1994). Effect of cold-exposure on rat organ blood flows. *Arch. Physiol.* 102, 55–59. 10.3109/13813459408996106 7516734

[B2] Adrizal A. Patterson P. H. Angel C. R. Markant A. (2019). Feeding broiler chicks diets containing keto- and hydroxy-acids: performance and carcass weight. *Poult. Sci.* 98 3818–3827. 10.3382/ps/pez091 30839093

[B3] Agrawal R. Hirpurkar S. D. Sannat C. Gupta A. K. (2016). Comparative study on immunoglobulin Y transfer from breeding hens to egg yolk and progeny chicks in different breeds of poultry. *Vet. World* 9 425–431. 10.14202/vetworld.2016.425-431 27182141 PMC4864487

[B4] Aguanta B. N. Fuller A. L. Milfort M. C. Williams S. M. Rekaya R. Aggrey S. E. (2018). Histologic effects of concurrent heat stress and coccidial infection on the lymphoid tissues of broiler chickens. *Avian Dis.* 62 345–350. 10.1637/11907-052818-reg.1 31119917

[B5] Alexander G. Bell A. W. Setchell B. P. (1972). Regional distribution of cardiac output in young lambs: effect of cold exposure and treatment with catecholamines. *J. Physiol.* 220 511–528. 10.1113/jphysiol.1972.sp009720 5016035 PMC1331667

[B6] Annison E. F. Hill K. J. Kenworthy R. (1968). Volatile fatty acids in the digestive tract of the fowl. *Br. J. Nutr.* 22 207–216. 10.1079/bjn19680026 5673541

[B7] Bai W. Q. Zhang K. Y. Ding X. M. Bai S. P. Wang J. P. Peng H. W. (2019). High dietary energy content increases inflammatory markers after lipopolysaccharide challenge in meat ducks. *Poult. Sci.* 98 164–171. 10.3382/ps/pey380 30137491

[B8] Bar A. Vax E. Hunziker W. Halevy O. Striem S. (1996). The role of gonadal hormones in gene expression of calbindin (Mr 28,000) in the laying hen. *Gen. Comp. Endocrinol.* 103 115–122. 10.1006/gcen.1996.0100 8812346

[B9] Barber D. L. Sanders E. J. Aebersold R. Schneider W. J. (1991). The receptor for yolk lipoprotein deposition in the chicken oocyte. *J. Biol. Chem.* 266 18761–18770. 1655760

[B10] Behrman R. E. Lees M. H. (1971). Organ flow of the fetal, newborn and adult rhesus monkey: a comparative study. *Biol. Neonate* 18 330–340. 10.1159/000240374 5005912

[B11] Benton M. J. Donoghue P. C. J. Asher R. J. Friedman M. Near T. J. Jakob V. (2015). Constraints on the timescale of animal evolutionary history. *Palaeontol. Electron.* 18.1.1FC 1–106. 10.1006/anbe.1999.1287 10640361

[B12] Bertzbach L. D. Laparidou M. Härtle S. Etches R. J. Kaspers B. Schusser B. (2018). Unraveling the role of B cells in the pathogenesis of an oncogenic avian herpesvirus. *Proc. Natl. Acad. Sci. U.S.A.* 115 11603–11607. 10.1073/pnas.1813964115 30337483 PMC6233081

[B13] Boa-Amponsem K. Larsen C. T. Dunnington E. A. Siegel P. B. (1999). Immunocompetence and resistance to marble spleen disease of broiler- and layer-type pure lines of chickens. *Avian Pathol.* 28 379–384. 10.1080/03079459994641 26905495

[B14] Boelkins J. N. Mueller W. J. Hall K. L. (1973). Cardiac output distribution in the laying hen during shell formation. *Comp. Biochem. Physiol. A* 46 735–743. 10.1016/0300-9629(73)90125-4 4127752

[B15] Brody T. B. Siegel P. B. Cherry J. A. (1984). Age, body weight and body composition requirements for the onset of sexual maturity of dwarf and normal chickens. *Br. Poult. Sci.* 25 245–252. 10.1080/00071668408454863 6733556

[B16] Brusatte S. L. O’Connor J. K. Jarvis E. D. (2015). The origin and diversification of birds. *Curr. Biol.* 25 R888–R898. 26439352 10.1016/j.cub.2015.08.003

[B17] Bujo H. Hermann M. Kaderli M. O. Jacobsen L. Sugawara S. Nimpf J. (1994). Chicken oocyte growth is mediated by an eight ligand binding repeat member of the LDL receptor family. *EMBO J.* 13 5165–5175. 10.1002/j.1460-2075.1994.tb06847.x 7957081 PMC395465

[B18] Buyse J. Adelsohn D. S. Decuypere E. Scanes C. G. (1993). Diurnal-nocturnal changes in food intake, gut storage of ingesta food transit time and metabolism in growing broiler chickens: a model for temporal control of energy balance. *Br. Poult. Sci.* 34 699–709. 10.1080/00071669308417628 8242406

[B19] Castillo L. Tanaka Y. Wineland M. J. Jowsey J. O. DeLuca H. F. (1979). Production of 1,25-dihydroxyvitamin D3 and formation of medullary bone in the egg-laying hen. *Endocrinology* 104 1598–1601. 10.1210/endo-104-6-1598 446379

[B20] Chen H. Huang R. L. Zhang H. X. Di K. Q. Pan D. Hou Y. G. (2007). Effects of photoperiod on ovarian morphology and carcass traits at sexual maturity in pullets. *Poult. Sci.* 86 917–920. 10.1093/ps/86.5.917 17435026

[B21] Chen Y.-H. Lee S.-M. Hsu J.-C. Chang Y.-C. Wang S.-Y. (2014). Methane generation from the intestine of Muscovy ducks, mule ducks and white Roman geese. *Aerosol Air Qual. Res.* 14 323–329. 10.4209/aaqr.2013.05.0180

[B22] Chen Y. H. Wang S. Y. Hsu J. C. (2003). Effects of caecectomy on body weight gain, intestinal characteristics and enteric gas production in goslings. *Asian Australas J. Anim. Sci.* 16 1030–1034. 10.5713/ajas.2003.1030

[B23] Clemens E. T. Stevens C. E. Southworth M. (1975). Sites of organic acid production and pattern of digesta movement in the gastrointestinal tract of geese. *J. Nutr.* 105 1341–1350. 10.1093/jn/105.10.1341 240009

[B24] de Beer M. McMurtry J. P. Brocht D. M. Coon C. N. (2008). An examination of the role of feeding regimens in regulating metabolism during the broiler breeder grower period. 2. Plasma hormones and metabolites. *Poult. Sci.* 87 264–275. 10.3382/ps.2007-00196 18212369

[B25] Deeley R. G. Mullinix D. P. Wetekam W. Kronenberg H. M. Meyers M. Eldridge J. D. (1975). Vitellogenin synthesis in the avian liver. Vitellogenin is the precursor of the egg yolk phosphoproteins. *J. Biol. Chem.* 250 9060–9066. 811661

[B26] Dunn I. C. Wilson P. W. Shi Y. Burt D. W. Loudon A. S. I. Sharp P. J. (2017). Diurnal and photoperiodic changes in thyrotrophin-stimulating hormone β expression and associated regulation of deiodinase enzymes (DIO2, DIO3) in the female juvenile chicken hypothalamus. *J. Neuroendocrinol.* 29:e12554. 10.1111/jne.12554 29117457 PMC5767736

[B27] Elkin R. G. Bauer R. Schneider W. J. (2012). The restricted ovulator chicken strain: an oviparous vertebrate model of reproductive dysfunction caused by a gene defect affecting an oocyte-specific receptor. *Anim. Reprod. Sci.* 136 1–13. 10.1016/j.anireprosci.2012.10.002 23123285 PMC3521959

[B28] Elkin R. G. Zhong Y. Porter R. E. Jr. Walzem R. L. (2003). Validation of a modified PCR-based method for identifying mutant restricted ovulator chickens: substantiation of genotypic classification by phenotypic traits. *Poult. Sci.* 82 517–525. 10.1093/ps/82.4.517 12710468

[B29] Emiola I. A. Ojebiyi O. O. Akande T. O. (2011). Performance and organ weights of laying hens fed diets containing graded levels of sun-dried cocoa bean shell (CBS). *Int. J. Poult. Sci.* 10 986–989.

[B30] Ewert D. L. Eidson C. S. (1977). Effect of bursectomy and depletion of immunoglobulin A on antibody production and resistance to respiratory challenge after local or systemic vaccination of chickens with Newcastle disease virus. *Infect. Immun.* 18 146–150. 10.1128/iai.18.1.146-150.1977 908616 PMC421206

[B31] Fallahsharoudi A. de Kock N. Johnsson M. Ubhayasekera S. J. Bergquist J. Wright D. (2015). Domestication effects on stress induced steroid secretion and adrenal gene expression in chickens. *Sci. Rep.* 5:15345. 10.1038/srep15345 26471470 PMC4608001

[B32] Fleming D. S. Weigend S. Simianer H. Weigend A. Rothschild M. Schmidt C. (2017). Genomic comparison of indigenous African and Northern European chickens reveals putative mechanisms of stress tolerance related to environmental selection pressure. *G3* 7 1525–1537. 10.1534/g3.117.041228 28341699 PMC5427493

[B33] Garland Flink L. Allen R. Barnett R. Malmström H. Peters J. Eriksson J. (2014). Establishing the validity of domestication genes using DNA from ancient chickens. *Proc. Natl. Acad. Sci. U.S.A.* 111 6184–6189. 10.1073/pnas.1308939110 24753608 PMC4035994

[B34] Gilbert A. B. (1971). “The egg: its physical and chemical aspects,” in *Physiology and Biochemistry of the Domestic Fowl*, Vol. 3 eds Bell D. J. Freeman B. M. (London: Academic Press), 1379–1399.

[B35] Glick B. Timothy S. Chang T. S. Jaap R. G. (1956). The Bursa of Fabricius and antibody production. *Poult. Sci.* 35 224–225. 10.3382/ps.0350224

[B36] Gloux A. Le Roy N. Ezagal J. Même N. Hennequet-Antier C. Piketty M. L. (2020). Possible roles of parathyroid hormone, 1.25(OH)2D3, and fibroblast growth factor 23 on genes controlling calcium metabolism across different tissues of the laying hen. *Domest. Anim. Endocrinol.* 72:106407. 10.1016/j.domaniend.2019.106407 32006872

[B37] Grajal A. Strahl S. D. Parra R. Gloria Dominguez M. Neher A. (1989). Foregut fermentation in the hoatzin, a neotropical leaf-eating bird. *Science* 245 1236–1238. 10.1126/science.245.4923.1236 17747887

[B38] Grau C. R. Roudybush T. E. McGibbon W. H. (1979). Mineral composition of yolk fractions and whole yolk from eggs of restricted ovulator hens. *Poult. Sci.* 58, 1143–1148. 10.3382/ps.0581143

[B39] Grozeva N. Valchev I. Binev R. Lazarov L. Hristov T. Kanakov D. (2017). Pathomorphological changes in the spleen of turkey broilers challenged with Aflatoxin B1 alone or co-administered with Mycotox NG. *Int. J. Vet. Sci. Technol.* 1 1–6.

[B40] Hamal K. R. Burgess S. C. Pevzner I. Y. Erf G. F. (2006). Maternal antibody transfer from dams to their egg yolks, egg whites, and chicks in meat lines of chickens. *Poult. Sci.* 85 1364–1372. 10.1093/ps/85.8.1364 16903465

[B41] Hassan M. R. Sultana S. Choe H. S. Ryo K. S. (2016). Effect of monochromatic and combined light colour on performance, blood parameters, ovarian morphology and reproductive hormones in laying hens. *Ital. J. Anim. Sci.* 12:3.

[B42] Havenstein G. B. Ferket P. R. Qureshi M. A. (2003). Growth, livability, and feed conversion of 1957 versus 2001 broilers when fed representative 1957 and 2001 broiler diets. *Poult. Sci.* 82 1500–1508. 10.1093/ps/82.10.1500 14601725

[B43] Havenstein G. B. Ferket P. R. Scheideler S. E. Larson B. T. (1994). Growth, livability, and feed conversion of 1957 vs 1991 broilers when fed “typical” 1957 and 1991 broiler diets. *Poult. Sci.* 73 1785–1794. 10.3382/ps.0731785 7877934

[B44] Hayes M. A. Wobeser G. A. (1983). Subacute toxic effects of dietary T-2 toxin in young mallard ducks. *Can. J. Comp. Med.* 47 180–187. 6883185 PMC1235915

[B45] Hippeläinen M. Naukkarinen A. (1990). Effects of local and systemic immunization on serum antibody titres in splenectomized chickens. *APMIS* 98 131–136. 10.1111/j.1699-0463.1990.tb01012.x 2302348

[B46] Hrabia A. Paczoska-Eliasiewicz H. Niezgoda J. Rząsa J. (2005). Histamine affects blood flow through the reproductive organs of the domestic hen (*Gallus domesticus*). *Folia Biol.* 53 209–213. 10.3409/173491605775142864 19058546

[B47] Ishise S. Pegram B. L. Yamamoto J. Kimamura Y. Frolich E. D. (1980). Reference sample microsphere method: cardiac output and blood flows in conscious rats. *Am. J. Physiol.* 239 H443–H449. 7425136 10.1152/ajpheart.1980.239.4.H443

[B48] Johannsen S. A. Rasmussen M. A. Hensley M. J. Wilhelms K. Griffith R. Scanes C. G. (2005). Effects of *Lactobacilli* and Lactose on *Salmonella typhimurium* Colonisation and Microbial Fermentation in the Crop of the Young Turkey. *Br. Poult. Sci.* 46 708–716. 16428114 10.1080/00071660500393694

[B49] John J. L. (1994). The avian spleen: a neglected organ. *Q. Rev. Biol.* 69 327–351. 10.1086/418649 7972679

[B50] Jones D. R. Bryan R. M. Jr. West N. H. Lord R. H. Clark B. (1979). Regional distribution of blood flow during diving in the duck (*Anas platyrhynchos*). *Can. J. Zool.* 57 995–1002. 10.1139/z79-126

[B51] Joseph N. S. Robinson F. E. Korver D. R. Renema R. A. (2000). Effect of dietary protein intake during the pullet-to-breeder transition period on early egg weight and production in broiler breeders. *Poult. Sci.* 79 1790–1796. 10.1093/ps/79.12.1790 11194042

[B52] Kannan T. A. Ramesh G. Ushakumari S. Dhinakarraj G. Vairamuthu S. (2015). Electron microscopic studies of spleen in chicken (*Gallus domesticus*). *J. Adv. Vet. Anim. Res.* 4 160–165.

[B53] Karlsson A. C. Svemer F. Eriksson J. Darras V. M. Andersson L. Jensen P. (2015). The effect of a mutation in the thyroid stimulating hormone receptor (TSHR) on development, behavior and TH levels in domesticated chickens. *PLoS One* 10:e0129040. 10.1371/journal.pone.0129040 26053744 PMC4460094

[B54] Kaul R. Gerstberger R. Meyer J. U. Simon E. (1983). Salt gland blood flow in saltwater-adapted Pekin ducks: microsphere measurement of the proportionality to secretion rate and investigation of controlling mechanisms. *J. Comp. Physiol.* 149 457–462. 10.1007/bf00690003

[B55] Khan S. A. Venancio E. J. Ono M. A. Fernandes E. V. Hirooka E. Y. Shimizu C. F. (2019). Effects of subcutaneous ochratoxin-A exposure on immune system of broiler chicks. *Toxins* 11:264. 10.3390/toxins11050264 31083513 PMC6563231

[B56] Khare V. M. Saxena V. K. Tomar A. Nyinawabera A. Singh K. B. Ashby C. R. Jr. (2019). Cytokine gene expression following RSV-A infection. *Front. Biosci.* 24 463–481. 10.2741/4729 30468667

[B57] Kinsky F. C. (1971). The consistent presence of paired ovaries in the Kiwi*(Apteryx)* with some discussion of this condition in other birds. *J. Ornithol.* 112 334–357. 10.1007/bf01640692

[B58] Ksepka D. T. Stidham T. A. Williamson T. E. (2017). Early Paleocene landbird supports rapid phylogenetic and morphological diversification of crown birds after the K-Pg mass extinction. *Proc. Natl. Acad. Sci. U.S.A.* 114 8047–8052. 10.1073/pnas.1700188114 28696285 PMC5544281

[B59] Kumar S. Chen C. Indugu N. Werlang G. O. Singh M. Kim W. K. (2018). Effect of antibiotic withdrawal in feed on chicken gut microbial dynamics, immunity, growth performance and prevalence of foodborne pathogens. *PLoS One* 13:e0192450. 10.1371/journal.pone.0192450 29444134 PMC5812630

[B60] Kwakkel R. P. Van Esch J. A. Ducro B. J. Koops W. J. (1995). Onset of lay related to multiphasic growth and body composition in White Leghorn pullets provided ad libitum and restricted diets. *Poult. Sci.* 74 821–832. 10.3382/ps.0740821 7603959

[B61] Laursen A. M. S. Kulkarni R. R. Taha-Abdelaziz K. Plattner B. L. Read L. R. Sharif S. (2018). Characterizaton of gamma delta T cells in Marek’s disease virus (Gallid herpesvirus 2) infection of chickens. *Virology* 522 56–64. 10.1016/j.virol.2018.06.014 30014858

[B62] Li G. Bronk J. T. Kelly P. J. (1989). Canine bone blood flow estimated with microspheres. *J. Orthop. Res.* 7 61–67. 10.1002/jor.1100070109 2908913

[B63] Li R. Lin J. Hou X. Han S. Weng H. Xu T. (2018). Characterization and roles of Cherry Valley Duck NLRP3 in innate immunity during avian pathogenic *Escherichia coli* infection. *Front. Immunol.* 9:2300. 10.3389/fimmu.2018.02300 30349536 PMC6186827

[B64] Loh T. C. Tan B. K. Foo H. L. Norhani A. Zulkifli I. (2011). Relationships of plasma and very low density lipoprotein lipids and subfractions with abdominal fat in chickens. *Asian Australas. J. Anim. Sci.* 24 82–87. 10.5713/ajas.2011.90622

[B65] Løtvedt P. Fallahshahroudi A. Bektic L. Altimiras J. Jensen P. (2017). Chicken domestication changes expression of stress-related genes in brain, pituitary and adrenals. *Neurobiol. Stress* 7 113–121. 10.1016/j.ynstr.2017.08.002 28879214 PMC5577413

[B66] Mansikka A. Jalkanen S. Sandberg M. Granfors K. Lassila O. Toivanen P. (1990). Bursectomy of chicken embryos at 60 hours of incubation leads to an oligoclonal B cell compartment and restricted Ig diversity. *J. Immunol.* 145 3601–3609. 2246505

[B67] Martínez Y. Carrión Y. Rodríguez R. Valdivié M. Olmo C. Betancur C. (2015). Growth performance, organ weights and some blood parameters of replacement laying pullets fed with increasing levels of wheat bran. *Rev. Bras. Cienc. Avic.* 17 347–353. 10.1590/1516-635x1703347-354

[B68] Matsumoto M. Kimura K. Fujisawa A. Matsuyama T. Asai T. Uyama O. (1982). Regional blood flows measured in Mongolian gerbil by a modified microsphere method. *Am. J. Physiol.* 42 H990–H995. 10.1152/ajpheart.1982.242.6.H990 7091359

[B69] Maurice D. V. Hughes B. L. Jones J. E. Weber J. M. (1982). The effect of reverse protein and low protein feeding regimens in the rearing period on pullet growth, subsequent performance, and liver and abdominal fat at end of lay. *Poult. Sci.* 61 2421–2429. 10.3382/ps.0612421 6897679

[B70] Mehaisen G. M. K. Eshak M. G. Elkaiaty A. M. Atta A.-R. M. M. Mashaly M. M. Abass A. O. (2017). Comprehensive growth performance, immune function, plasma biochemistry, gene expressions and cell death morphology responses to a daily corticosterone injection course in broiler chickens. *PLoS One* 12:e0172684. 10.1371/journal.pone.0172684 28235061 PMC5325522

[B71] Merrill G. F. Russo R. E. Halper J. M. (1981). Cardiac output distribution before and after endotoxin challenge in the rooster. *Am. J. Physiol.* 241 R67–R71. 7018271 10.1152/ajpregu.1981.241.1.R67

[B72] Miao L. P. Yuan C. Dong X. Y. Zhang X. Y. Zhou M. Y. Zou X. T. (2017). Effects of dietary L-arginine levels on small intestine protein turnover and the expression of genes related to protein synthesis and proteolysis of layers. *Poult. Sci.* 96, 1800–1808. 10.3382/ps/pew471 28340042

[B73] Miseta A. Bogner P. Berényi E. Kellermayer M. Galambos C. Wheatley D. N. (1993). Relationship between cellular ATP, potassium, sodium and magnesium concentrations in mammalian and avian erythrocytes. *Biochim. Biophys. Acta* 1175 133–139. 10.1016/0167-4889(93)90015-h 8418892

[B74] Moynihan J. B. Edwards N. A. (1975). Blood flow in the reproductive tract of the domestic hen. *Comp. Biochem. Physiol.* 51A, 745–748. 10.1016/0300-9629(75)90050-x 237694

[B75] Nassar F. S. El-Komy E. M. Abdou A. M. (2017). Ovarian morphology and egg quality traits of Egyptian selected strain for egg production compared with commercial laying strains. *Braz. J. Poult. Sci.* 19 683–688. 10.1590/1806-9061-2016-0455

[B76] Neutze J. M. Wyler F. Rudolph A. M. (1968). Use of radioactive microspheres to assess distribution of cardiac output in rabbits. *Am. J. Physiol.* 215 486–495. 10.1152/ajplegacy.1968.215.2.486 4874762

[B77] Niezgoda J. Bobek S. Kacińska M. (1979). Blood flow in the reproductive tract of the domestic hen following treatment with a pituitary gonadotropic inhibitor. *Acta Physiol. Pol.* 30 393–397. 495145

[B78] Nii T. Isobe N. Yoshimura Y. (2018). Effects of interleukin-1β and -6 on the expression of ion transporters involved in eggshell mineralization in cultured hen uterine mucosal tissue. *J. Poult. Sci.* 55 142–149. 10.2141/jpsa.0170138 32055167 PMC6756487

[B79] Nys Y. Baker K. Bouillon R. Van Baelen H. Lawson D. E. (1992). Regulation of calbindin D 28K and its mRNA in the intestine of the domestic hen. *Gen. Comp. Endocrinol.* 86 460–468. 10.1016/0016-6480(92)90071-q 1398006

[B80] Nys Y. Mayel-Afshar S. Bouillon R. Van Baelen H. Lawson D. E. (1989). Increases in calbindin D 28K mRNA in the uterus of the domestic fowl induced by sexual maturity and shell formation. *Gen. Comp. Endocrinol.* 76 322–329. 10.1016/0016-6480(89)90164-0 2591722

[B81] Oláh I. Glick B. (1982). Splenic white pulp and associated vascular channels in chicken spleen. *Am. J. Anat.* 165 445–480. 10.1002/aja.1001650408 7158614

[B82] Ossa J. E. Alexander J. Schurig G. G. (1983). Role of splenectomy in prevention of hemorrhagic enteritis and death from hemorrhagic enteritis virus in turkeys. *Avian Dis.* 27 1106–1111. 10.2307/1590210 6316896

[B83] Peltonen L. M. Sankari S. (2011). Ott’s protein osmotic pressure of serum and interstitial fluid in chickens (*Gallus gallus*): effect of age and gender. *J. Exp. Biol.* 214 599–606. 10.1242/jeb.048769 21270308

[B84] Pishnamazi A. Renema R. A. Zuidhof M. J. Robinson F. (2014). Effect of age at photostimulation on sexual maturation in broiler breeder pullets. *Poult. Sci.* 93 1274–1281. 10.3382/ps.2012-02834 24795323

[B85] Powers L. V. (2000). The avian spleen: anatomy, physiology and diagnostics. *Compend. Contin. Educ. Pract. Vet.* 22 838–843.

[B86] Ran J. S. Jin J. Zhang X. X. Wang Y. Ren P. Li J. J. (2018). Molecular characterization, expression and functional analysis of chicken *STING*. *Int. J. Mol. Sci.* 19:E3706. 10.3390/ijms19123706 PMC6321155 30469505

[B87] Ren Z. Piepenburg A. J. Yang X. Cook M. E. (2019). Effect of anti-fibroblast growth factor 23 antibody on phosphate and calcium metabolism in adenine gavaged laying hens. *Poult. Sci.* 98 4896–4900. 10.3382/ps/pez239 31064011

[B88] Roth L. S. Lind O. (2013). The impact of domestication on the chicken optical apparatus. *PLoS One* 8:e65509. 10.1371/journal.pone.0065509 23776492 PMC3680433

[B89] Rubin C.-J. Zody M. C. Eriksson J. Meadows J. R. S. Sherwood E. Webster M. T. (2010). Whole-genome resequencing reveals loci under selection during chicken domestication. *Nature* 464 587–591. 10.1038/nature08832 20220755

[B90] Rząsa J. Hrabia A. Paczoska-Eliasiewicz H. Sechman A. (2008). Changes in blood flow through the chicken ovary and oviduct after serotonin treatment. *Bull. Vet. Inst. Pulawy* 52 241–244.

[B91] Sajewicz-Krukowska J. Olszewska-Tomczyk M. Domańska-Blicharz K. (2017). *In ovo* administration of CpG ODN induces expression of immune response genes in neonatal chicken spleen. *J. Vet. Res.* 61 451–458. 10.1515/jvetres-2017-0050 29978109 PMC5937344

[B92] Sakanashi T. M. Brigham H. E. Rasmussen K. M. (1987). Effect of dietary restriction during lactation on cardiac output, organ blood flow and organ weights of rats. *J. Nutr.* 117 1469–1474. 10.1093/jn/117.8.1469 3625319

[B93] Saki A. A. Aliarabi H. Siyar S. A. H. Salari J. Hashemi M. (2014). Effect of a phytogenic feed additive on performance, ovarian morphology, serum lipid parameters and egg sensory quality in laying hen. *Vet. Res. Forum* 5 287–293. 25610580 PMC4299994

[B94] Sapirstein I. A. Hartman F. A. (1959). Cardiac output and its distribution in the chicken. *Am. J. Physiol.* 196 751–752. 10.1152/ajplegacy.1959.196.4.751 13637206

[B95] Sawai H. Kim H. L. Kuno K. Suzuki S. Gotoh H. Takada M. (2010). The origin and genetic variation of domestic chickens with special reference to junglefowls *Gallus g. gallus* and *G. varius*. *PLoS One* 5:e10639. 10.1371/journal.pone.0010639 20502703 PMC2873279

[B96] Scanes C. G. (2015). “Blood,” in *Sturkie’s Avian Physiology*, 6th Edn, ed. Scanes C. G. (Amsterdam: Elsevier), 167–191.

[B97] Scanes C. G. Campbell R. Griminger P. (1987). Control of energy balance during egg production in the laying hen. *J. Nutr.* 117 605–611. 10.1093/jn/117.3.605 3572573

[B98] Scanes C. G. Mozolic H. Kavanagh E. Merrill G. Rabii J. (1982). Distribution of blood flow in the ovary of control and prostaglandin F2a treated domestic fowl (*Gallus domesticus*)”. *J. Reprod. Fertil.* 64 227–231. 10.1530/jrf.0.0640227 6798212

[B99] Schuster M. K. Schmierer B. Shkumatava A. Kuchler K. (2004). Activin A and follicle-stimulating hormone control tight junctions in avian granulosa cells by regulating occludin expression. *Bill. Reprod.* 70 1493–1499. 10.1095/biolreprod.103.024331 14736813

[B100] Steyrer E. Barber D. L. Schneider W. J. (1990). Evolution of lipoprotein receptors. The chicken oocyte receptor for very low density lipoprotein and vitellogenin binds the mammalian ligand apolipoprotein E. *J. Biol. Chem.* 265 19575–19581. 2174044

[B101] Stifani S. Barber D. L. Nimpf J. Schneider W. J. (1990). A single chicken oocyte plasma membrane protein mediates uptake of very low density lipoprotein and vitellogenin. *Proc. Natl. Acad. Sci. U.S.A.* 87 1955–1959. 10.1073/pnas.87.5.1955 2308956 PMC53603

[B102] Stonerock R. H. Roland D. A. Sr. Voitle R. A. (1975). The effect of cropectomy on selected reproductive and physiological characteristics of laying hens. *Poult. Sci.* 54 288–294. 10.3382/ps.0540288 1135132

[B103] Sun C. Lu J. Yi G. Yuan J. Duan Z. Qu L. (2015). Promising loci and genes for yolk and ovary weight in chickens revealed by a genome-wide association study. *PLoS One* 10:e0137145. 10.1371/journal.pone.0137145 26332579 PMC4558091

[B104] Sun J. M. Richards M. P. Rosebrough R. W. Ashwell C. M. McMurtry J. P. Coon C. N. (2006). The relationship of body composition, feed intake, and metabolic hormones for broiler breeder females. *Poult. Sci.* 85 1173–1184. 10.1093/ps/85.7.1173 16830857

[B105] Sun X. Liu E. Wang T. Zhang Q. Yang P. Ahmed N. (2019). The novel histological evidence of the blood-spleen barrier in duck (*Anas platyrhynchos*). *Histol. Histopathol.* 34 33–45. 29971756 10.14670/HH-18-019

[B106] Takeuchi S. Takahashi S. (1998). Melanocortin receptor genes in the chicken-tissue distributions. *Gen. Comp. Endocrinol.* 112 220–231. 10.1006/gcen.1998.7167 9784305

[B107] Tan W. P. Riggs K. W. Thies R. L. Rurak D. W. (1997). Use of an automated fluorescent microsphere method to measure regional blood flow in the fetal lamb. *Can. J. Physiol. Pharmacol.* 75 959–968. 10.1139/y97-120 9360009

[B108] Thein E. Becker M. Anetzberger H. Hammer C. Messmer K. (2003). Direct assessment and distribution of regional portal blood flow in the pig by means of fluorescent microspheres. *J. Appl. Physiol.* 95 1808–1816. 10.1152/japplphysiol.00362.2003 12819221

[B109] Undar A. Masai T. Yang S. Q. Goddard-Finegold J. Frazier O. H. Fraser C. D. Jr. (1999). Effects of perfusion mode on regional and global organ blood flow in a neonatal piglet model. *Ann. Thorac. Surg.* 68 1336–1342. 10.1016/s0003-4975(99)00913-3 10543503

[B110] Wang P. Ba Z. F. Burkhardt J. Chaudry I. H. (1993). Trauma-hemorrhage and resuscitation in the mouse - effects on cardiac output and organ blood-flow. *Am. J. Physiol.* 264 H1166–H1173. 8476095 10.1152/ajpheart.1993.264.4.H1166

[B111] Wang S.-Y. Huang D.-J. (2005). Assessment of greenhouse gas emissions from poultry enteric fermentation. *Asian Aust. J. Anim. Sci.* 18 873–878. 10.5713/ajas.2005.873

[B112] West B. Zhou B.-X. (1988). Did chickens go north? New evidence for domestication. *J. Archaeol. Sci.* 15 515–533. 10.1016/0305-4403(88)90080-5

[B113] White D. H. Finley M. T. Ferrell J. F. (1978). Histopathologic effects of dietary cadmium on kidneys and testes of mallard ducks. *J. Toxicol. Environ. Health* 4 551–558. 10.1080/15287397809529678 682206

[B114] Wright A. D. Northwood K. S. Obispo N. E. (2009). Rumen-like methanogens identified from the crop of the folivorous South American bird, the hoatzin (*Opisthocomus hoazin*). *ISME J.* 3 1120–1126. 10.1038/ismej.2009.41 19387486

[B115] Wright D. Boije H. Meadows J. R. Bed’hom B. Gourichon D. Vieaud A. (2009). Copy number variation in Dintron 1 of SOX5 causes the Pea-comb phenotype in chickens. *PLoS Genet.* 5:e1000512. 10.1371/journal.pgen.1000512 19521496 PMC2685452

[B116] Xiang B. You R. Kang Y. Xie P. Zhu W. Sun M. (2019). Host immune responses of pigeons infected with Newcastle disease viruses isolated from pigeons. *Microb. Pathog.* 127 131–137. 10.1016/j.micpath.2018.11.049 30508624

[B117] Xiong D. Song L. Pan Z. Jiao X. (2018). Molecular cloning, characterization, and functional analysis of pigeon (*Columba livia*) Toll-like receptor 5. *Poult. Sci.* 97 4031–4039. 10.3382/ps/pey244 29945253

[B118] Yang J. H. Zhao Z. H. Hou J. F. Zhou Z. L. Deng Y. F. Dai J. J. (2013). Expression of TRPV6 and CaBP-D28k in the egg shell gland (uterus) during the oviposition cycle of the laying hen. *Br. Poult. Sci.* 54 398–406. 23796121 10.1080/00071668.2013.791385

[B119] Yang L. Liu G. Zhu X. Luo Y. Shang Y. Gu X. L. (2019). The anti-inflammatory and antioxidant effects of leonurine hydrochloride after lipopolysaccharide challenge in broiler chicks. *Poult. Sci.* 98 1648–1657. 10.3382/ps/pey532 30476291

[B120] Youssef A. A. El-Hamid A. E.-H. E. Abedalla A. A. Berika M. A. Al-Harthi M. A. Kucuk O. (2016). Laying performance, digestibility and plasma hormones in laying hens exposed to chronic heat stress as affected by betaine, vitamin C, and/or vitamin E supplementation. *Springer Plus* 5:1619. 10.1186/s40064-016-3304-0 PMC5028346 27652192

[B121] Zhang C. Geng Z. Y. Chen K. K. Zhao X. H. Wang C. (2019). L-theanine attenuates transport stress-induced impairment of meat quality of broilers through improving muscle antioxidant status. *Poult. Sci.* 98 4648–4655. 10.3382/ps/pez164 30951605

[B122] Zhang Y. Cui N. Han N. Wu J. Cui Z. Su S. (2017). Depression of vaccinal immunity to Marek’s disease by infection with chicken infectious anemia virus. *Front. Microbiol.* 8:1863. 10.3389/fmicb.2017.01863 29018431 PMC5622928

[B123] Zuidhof M. J. Schneider B. L. Carney V. L. Korver D. R. Robinson F. E. (2014). Growth, efficiency, and yield of commercial broilers from 1957, 1978, and 2005. *Poult. Sci.* 93 2970–2982. 10.3382/ps.2014-04291 25260522 PMC4988556

